# Single-cell transcriptomics reveals predominantly inflammatory endothelial cell responses and suppressed vascular repair in silicosis

**DOI:** 10.3389/fimmu.2025.1629226

**Published:** 2025-09-03

**Authors:** Dongli Cao, Huashun Cui, Zequan Chen, Heyang Li, Bing Li, Jianhua Wang

**Affiliations:** ^1^ Joint Research Center for Occupational Medicine and Health of Institute of Health and Medicine (IHM), Anhui University of Science and Technology, Huainan, China; ^2^ School of Medicine, Anhui University of Science and Technology, Huainan, China; ^3^ Anhui Province Engineering Laboratory of Occupational Health and Safety, Huainan, China; ^4^ School of Public Health, Anhui University of Science and Technology, Hefei, China; ^5^ Cancer Institute, Fudan University Shanghai Cancer Center, Fudan University, Shanghai, China

**Keywords:** silicosis, endothelial cells, coal dust, mouse model, single-cell transcriptomics

## Abstract

**Introduction:**

Silicosis is a progressive fibrotic lung disease without effective treatment options, and its pathogenesis remains incompletely understood, particularly the role of endothelial cells (ECs).

**Methods:**

Here, we utilized single-cell RNA sequencing to characterize endothelial responses in lungs from silica-exposed mice.

**Results:**

We identified two functionally distinct endothelial subpopulations: 1. An inflammatory EC subtype, exhibiting significantly increased abundance and characterized by high expression of neutrophil-recruiting factors such as Spp1 (osteopontin), CCL (C-C motif chemokine ligand), and ESAM (endothelial cell–selective adhesion molecule), suggesting active involvement in neutrophil influx and persistent inflammation. 2. A reparative EC subtype, marked by upregulation of angiogenesis and vascular repair pathways, which exhibited decreased abundance and functional suppression within the silicotic lung microenvironment.

**Discussion:**

These results indicate a pathological shift toward inflammation-amplifying endothelial cells and impaired reparative capacity during silicosis progression. Our findings provide new mechanistic insights into endothelial cell dysfunction in silicosis and highlight potential targets for therapeutic intervention.

## 1 Introduction

Silicosis is a chronic occupational lung disease characterized by persistent lung inflammation and progressive fibrosis caused by long-term inhalation of crystalline silica particles (1). In the silicotic lung, inhaled silica particles trigger a robust inflammatory response and lead to the formation of fibrotic nodules, resulting in irreversible damage to the lung architecture (2). Despite its global prevalence and severity, there are currently no effective therapies to halt or reverse silicosis, which highlights the need to better understand its pathogenesis and identify new therapeutic targets.

The development of silicosis involves a complex interplay of various cell types, including macrophages, epithelial cells, fibroblasts, and endothelial cells, all contributing to chronic inflammation and aberrant tissue remodeling (3). ECs, which form the inner lining of blood vessels, have traditionally been viewed as passive barriers, but emerging evidence indicates they play active roles in regulating inflammation and fibrosis. For example, ECs can produce pro-inflammatory cytokines and express adhesion molecules that facilitate leukocyte recruitment, and they can undergo endothelial-to-mesenchymal transition (EndMT) to transform into fibroblast-like cells, potentially contributing directly to fibrotic scar formation (4). Conversely, endothelial cells are also crucial for tissue repair and regeneration, as they participate in angiogenesis and help restore the blood supply to injured tissue (5). However, the specific roles of endothelial cells in silicosis, which are whether they predominantly exacerbate lung injury, aid in repair, or both, remain poorly understood.

In this study, we employed a well-established murine model of silicosis combined with single-cell RNA sequencing (scRNA-seq) to investigate EC responses comprehensively. Our goal was to characterize endothelial behavior at the cellular and molecular levels in silicotic lungs, specifically identifying distinct EC subpopulations associated with either inflammatory or reparative functions. By elucidating these differential roles, we aim to enhance understanding of silicosis pathogenesis and facilitate the development of targeted therapeutic strategies for endothelial dysfunction.

## 2 Materials and methods

### 2.1 Single-cell RNA sequencing analysis

We collected single-cell RNA sequencing data in matrix format from the GEO database and subsequently imported the gene expression matrix into R software (version 4.0.0). For downstream analysis, we utilized the Seurat package (version 4.0.0). The gene expression matrix underwent normalization and scaling procedures for each sample. Genes expressed in more than 10 cells were retained for further analysis. Cells with a high proportion (≥10%) of unique molecular identifiers (UMIs) mapping to mitochondrial genes were excluded, as well as cells with fewer than 200 detected genes. We employed the DoubletFinder function for doublet detection and filtering of cells. Following data integration, the expression levels of highly variable genes were scaled and centered.

Clusters were identified using the FindClusters function in Seurat, with a resolution parameter set to 0.1. Differential expression analysis between the identified clusters was performed using the FindAllMarkers function.

### 2.2 Cell type annotation

Marker genes were identified through the FindAllMarkers function, and cell types were annotated based on well-established classical markers. A second round of clustering was conducted using the same parameters to identify subclusters within the major cell types.

### 2.3 Trajectory analysis

Trajectory analyses were carried out using Monocle2 to predict differentiation pathways among endothelial subclusters.

### 2.4 Cell-cell communication analysis

The CellChat R package (version 1.1.3) was utilized to infer the intercellular communication network between clusters. A CellChat object was created with the normalized expression matrix, and visualization was performed using functions such as netVisual_aggregate and netAnalysis_contribution.

### 2.5 Animals

All experimental procedures were approved by the Institutional Animal Care and Use Committee of Anhui University of Science and Technology (Approval No. 20240301) and conducted in accordance with the ARRIVE guidelines. Male C57BL/6 mice (6–8 weeks old, 20–22 g) were purchased from Cavens Laboratory Animal Co., Ltd (Suzhou, China) and housed under specific pathogen-free conditions with a 12 h light/dark cycle, ambient temperature (22 ± 1°C), and ad libitum access to food and water.

### 2.6 Silica-induced pulmonary fibrosis model

The silicosis mouse model was established following the protocol described by Li et al. with modifications. Briefly, mice were randomly divided into control (n=20) and silica-exposed groups (n=20). After anesthesia with 3% isoflurane, animals received a single intranasal instillation of 50 μl sterile silica suspension (Sigma-Aldrich; Sigma, 14808-60-7; SiO_2_ particles, 1–5 μm diameter, 100 mg/ml in saline). Control mice received equal-volume saline instillation. To investigate dynamic pathological changes, subgroups of silica-exposed mice (n=10 per timepoint) were euthanized at 7 days (acute inflammation phase) and 56 days (fibrotic phase) post-exposure via intraperitoneal pentobarbital overdose (150 mg/kg).

### 2.7 Lung tissue processing for paraffin embedding

Lung tissues were fixed via tracheal cannulation with 4% paraformaldehyde under room temperature for 24 h, followed by graded ethanol dehydration (70% to 100%), xylene clearing, and paraffin infiltration (60°C, two cycles). Paraffin-embedded blocks were sectioned at 5 μm thickness using a rotary microtome (Leica RM2235).

### 2.8 Immunofluorescence staining

Paraffin-embedded lung tissue sections (4-μm thickness) were deparaffinized in xylene and rehydrated through a graded ethanol series (100%, 95%, 70%). Antigen retrieval was performed by boiling slides in 10 mM sodium citrate buffer (pH 6.0) for 20 min using a microwave. After cooling to room temperature (RT), sections were permeabilized with 0.3% Triton X-100 for 15 min and blocked with 5% normal serum (species-matched to secondary antibodies) containing 1% BSA for 1 h at RT. Tissues were incubated overnight at 4°C with primary antibodies against VEGF-a (Servicebio, Cat. No. GB15165-100; 1:200; green fluorescence) and HIF-1α (36169S; 1:200; red fluorescence), followed by species-specific Alexa Fluor-conjugated secondary antibodies (Servicebio, Cat. No. G1213-100UL; 1:500; 1 h at RT). Nuclei were counterstained with DAPI (0.1 μg/mL, 10 min). Slides were mounted with antifade medium (ProLong Gold) and imaged using a confocal laser scanning microscope (FV2000, Olympus) with a 20× or 40× oil-immersion objective. Co-localization analysis of VEGF-a and HIF-1α signals was performed using ImageJ (Fiji plugin) with threshold-based region-of-interest (ROI) selection.

### 2.9 Statistical analysis

**Figure 1:**
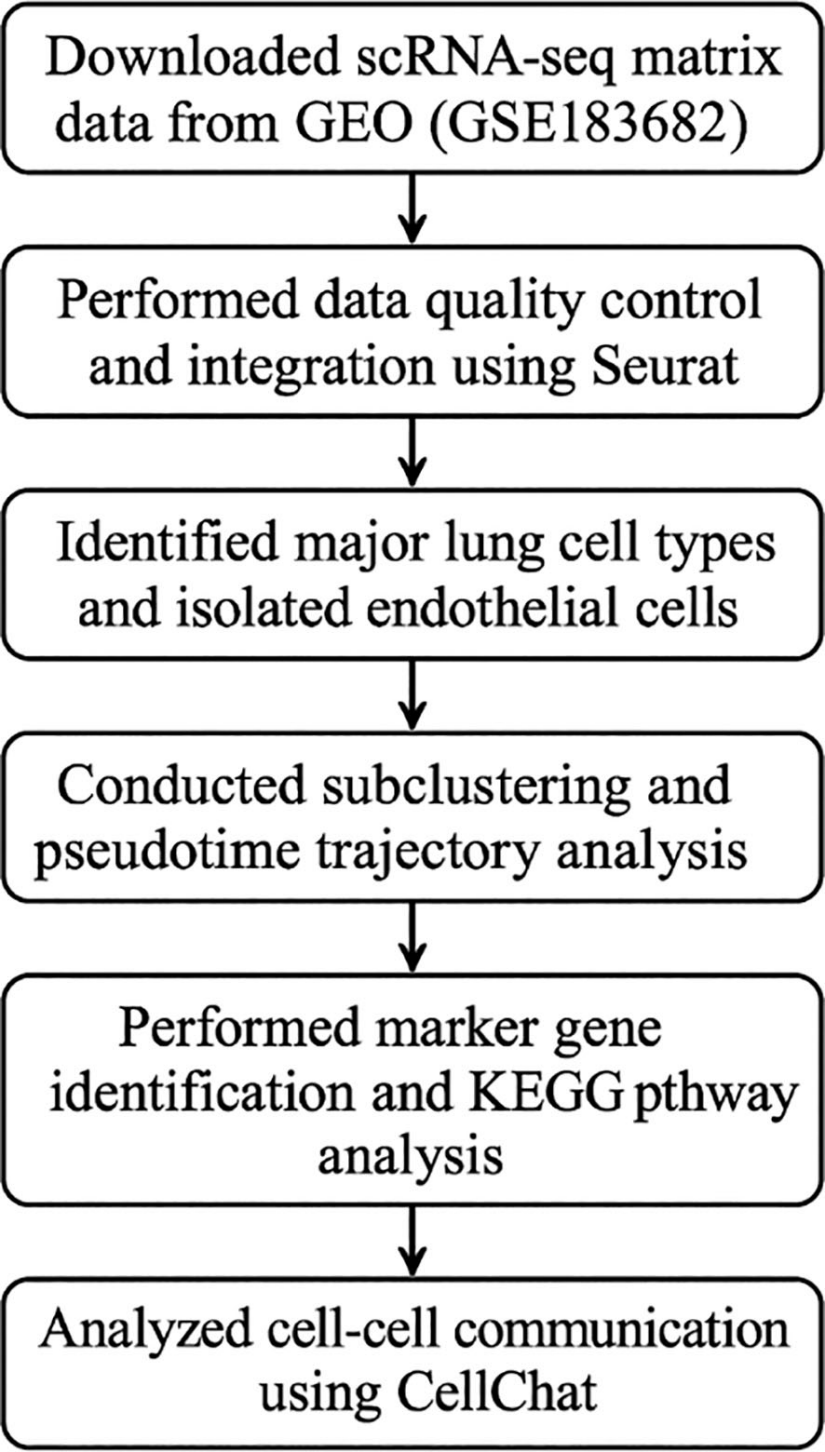
Workflow of scRNA-seq analysis in silicosis.

Quantitative data for VEGF-a and HIF-1α fluorescence intensity (Panels G-H) are expressed as mean ± standard deviation (SD). Statistical significance between groups was assessed using unpaired two-tailed Student’s t-test (for two-group comparisons) or one-way ANOVA with Tukey’s *post hoc* test (for multi-group comparisons). Normality and variance homogeneity were verified using Shapiro-Wilk and Levene’s tests, respectively. Sample size (n) represents biologically independent lung tissue sections (technical replicates: 3–5 fields per section). Significance thresholds were defined as *p < 0.05 and **p < 0.01; ns indicates non-significant differences (p ≥ 0.05). All analyses were conducted using GraphPad Prism v9.0 (**Figure 1**).

## 3 Results

### 3.1 Single-cell analysis reveals endothelial cell subpopulation shifts in a murine silicosis model

**Figure 2:**
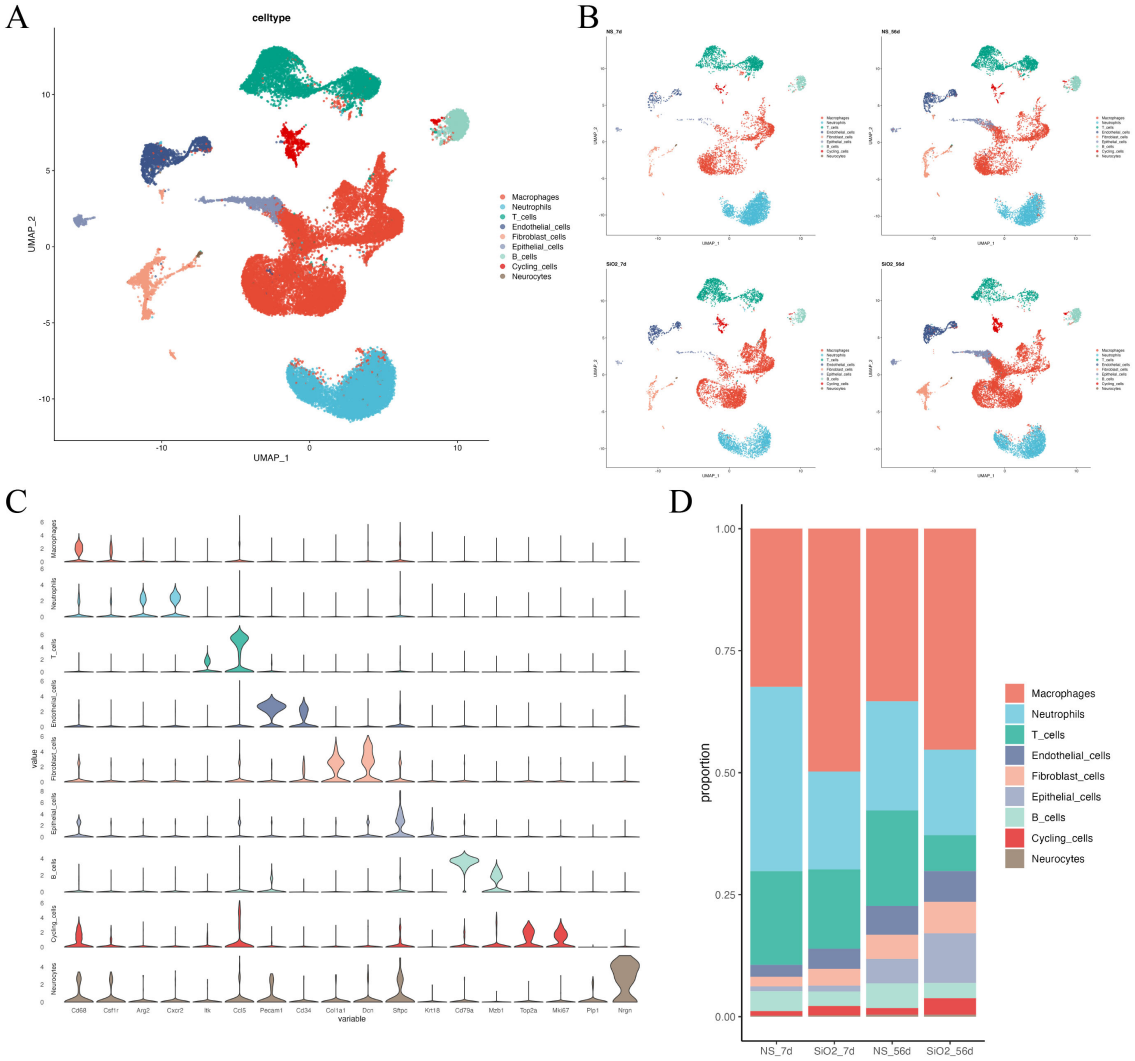
Single-cell RNA sequencing analysis of lung tissues in SiO_2_-induced mouse models. **(A)** UMAP plot showing major cell types, including macrophages, neutrophils, T cells, B cells, endothelial cells, epithelial cells, fibroblasts, cycling cells, and neuronal cells. **(B)** UMAP plots of cell-type distributions across different groups (NS-7d, SiO_2_-7d, NS-56d, SiO_2_-56d). **(C)** Violin plots showing the expression of marker genes in different cell populations. **(D)** Bar plot comparing cell type proportions across groups.

To investigate the potential mechanisms of endothelial cells during the progression of silicosis, we analyzed scRNA-Seq data from lung tissues of SiO2-7d and SiO2-56d silica-induced mouse models (6). Transcriptomic data from all groups were integrated and normalized, followed by non-linear clustering using UMAP. Cell clusters were annotated using classical cell markers from the CellMarker database, identifying nine major cell types: macrophages, neutrophils, T cells, B cells, endothelial cells, epithelial cells, fibroblasts, cycling cells, and neuronal cells, which were visualized on the UMAP plot (**Figures 2A–C**). To explore the cellular composition across samples, we calculated the proportion of each cell type in each group (**Figure 2D**, **Supplementary Table S1**). Compared to control mice (NS-7d), silica-exposed mice at 7 days (SiO_2_-7d) exhibited a significantly increased macrophage proportion (from approximately 30% to nearly 50%), consistent with Zhou et al. who highlighted the predominant role of macrophages in early inflammation. Notably, the proportion of endothelial cells was also significantly increased at both 7 days and 56 days post-exposure (NS-56d: 3%; SiO_2_-56d: 6%), indicating sustained endothelial activation throughout silicosis progression.

### 3.2 Macrophages mediate endothelial cell migration and depletion

**Figure 3:**
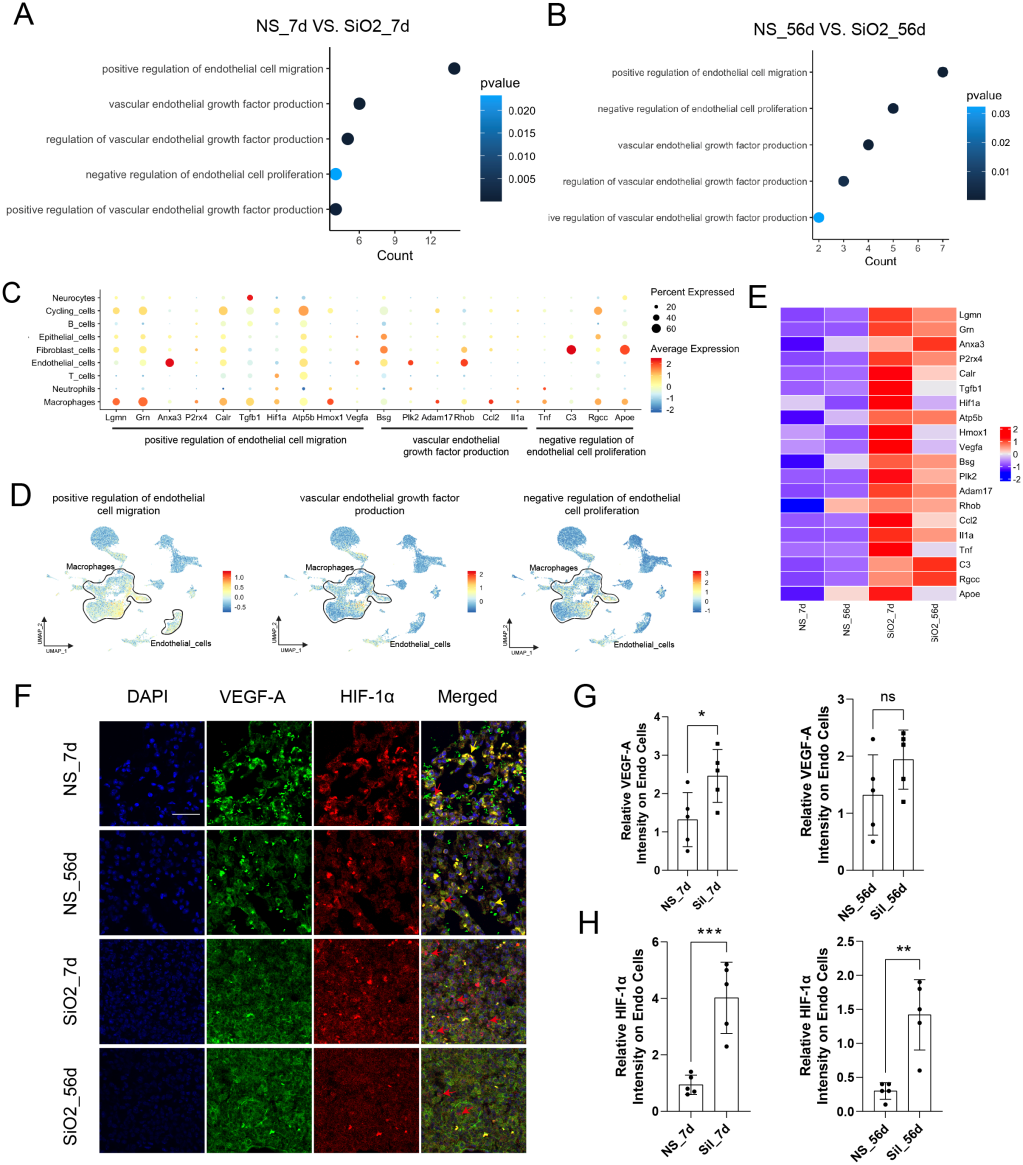
Macrophages influence endothelial cell migration and depletion. **(A, B)** GO enrichment analysis of DEGs in the 7d **(A)** and 56d **(B)** groups. **(C)** Dot plot displaying marker gene expression in various cell types. **(D)** UMAP feature plots highlighting endothelial cell-related biological processes, with macrophage-associated signals marked. **(E)** Heatmap showing key DEGs across different groups. **(F)** Confocal images of VEGF-A (green), HIF-1α (red), and nuclei (DAPI, blue) co-localization in endothelial cells. Scale bars: 50 μm (overview). Arrowheads indicate co-localized VEGF-A and HIF-1α signals. Red arrows highlight migrating endothelial cells; yellow arrows mark stationary cells. **(G, H)** Quantification of relative fluorescence intensity of VEGF-A **(G)** and HIF-1α **(H)** in endothelial cells. Data are mean ± SD (n indicated in figure). Statistical tests: *p<0.05, **p<0.01, ***p<0.001, ns: not significant.

We performed differential gene expression (DEG) and Gene Ontology (GO) enrichment analyses at 7 and 56 days to investigate the molecular processes underlying endothelial changes. GO analysis revealed significant enrichment of endothelial cell migration, vascular endothelial growth factor production, and negative regulation of endothelial proliferation (**Figures 3A, B**). Heatmap and dot plot analyses further demonstrated enhanced expression of inflammatory and pro-angiogenic genes such as Vegfa, Hif-1α, Tnf, II1a, and Apoe, predominantly within macrophages and endothelial cells (**Figures 3C, E**). Feature plots corroborated these findings, illustrating macrophage-driven regulation of endothelial biological processes, including migration and angiogenesis (**Figure 3D**).

Immunofluorescence staining validated these bioinformatics results, showing significantly increased co-localization of VEGF-a and HIF-1α proteins in endothelial cells following silica exposure (**Figure 3F**). Quantitative analysis indicated significantly higher VEGF-a expression in endothelial cells at 7 days post-silica exposure compared to controls (p < 0.05), whereas no significant difference was observed at 56 days (**Figure 3G**). In contrast, HIF-1α expression was markedly elevated in endothelial cells at both 7 days (p < 0.001) and 56 days (p < 0.01) post-exposure compared to controls (**Figure 3H**). These findings confirm a key role for macrophage-mediated VEGF-a and HIF-1α signaling in endothelial cells during silica-induced lung injury.

### 3.3 Diversity and functional analysis of endothelial cell subpopulations

**Figure 4:**
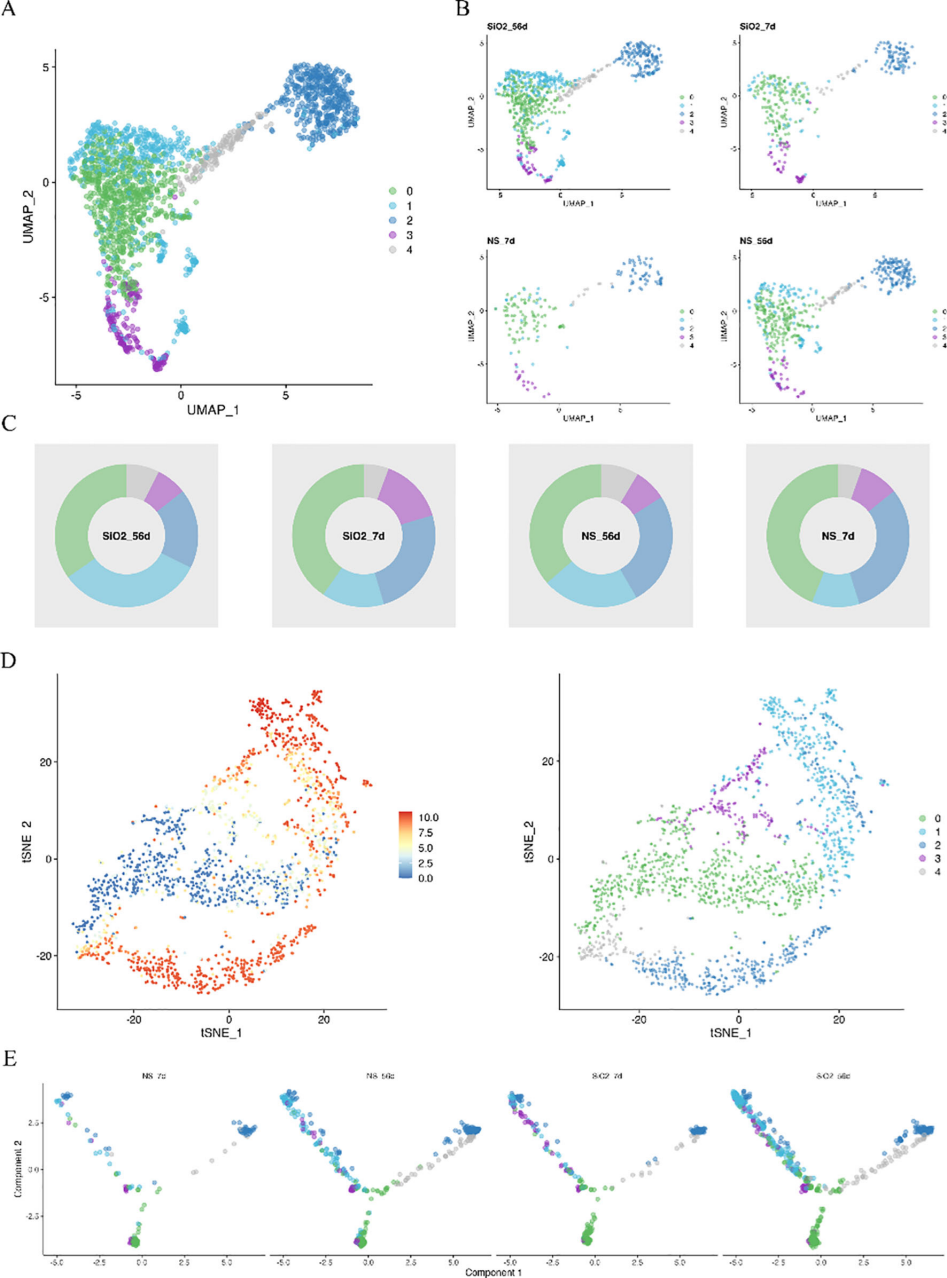
Endothelial cell heterogeneity and differentiation trajectories. **(A, B)** Clustering analysis identifies five endothelial cell subpopulations. **(C)** Proportion analysis of endothelial subpopulations across different groups. **(D, E)** Pseudotime trajectory analysis tracing endothelial cell differentiation from the C0 subpopulation toward C1 and C2.

To further dissect endothelial heterogeneity, we subclustered endothelial cells into five distinct subpopulations (C0–C4) (**Figures 4A, B**). The composition of endothelial subpopulations shifted notably following silica exposure; specifically, the proportion of subpopulation C1 significantly increased (from 21.8% to 32.8%) at 56 days, whereas subpopulations C0 and C2 decreased, suggesting divergent differentiation trajectories associated with silicosis progression (**Figure 4C**). Pseudotime trajectory analyses supported this bifurcation, revealing two primary differentiation paths from progenitor-like (C0) cells towards pro-inflammatory (C1) and angiogenesis-related (C2) states (**Figures 4D, E**).

**Figure 5:**
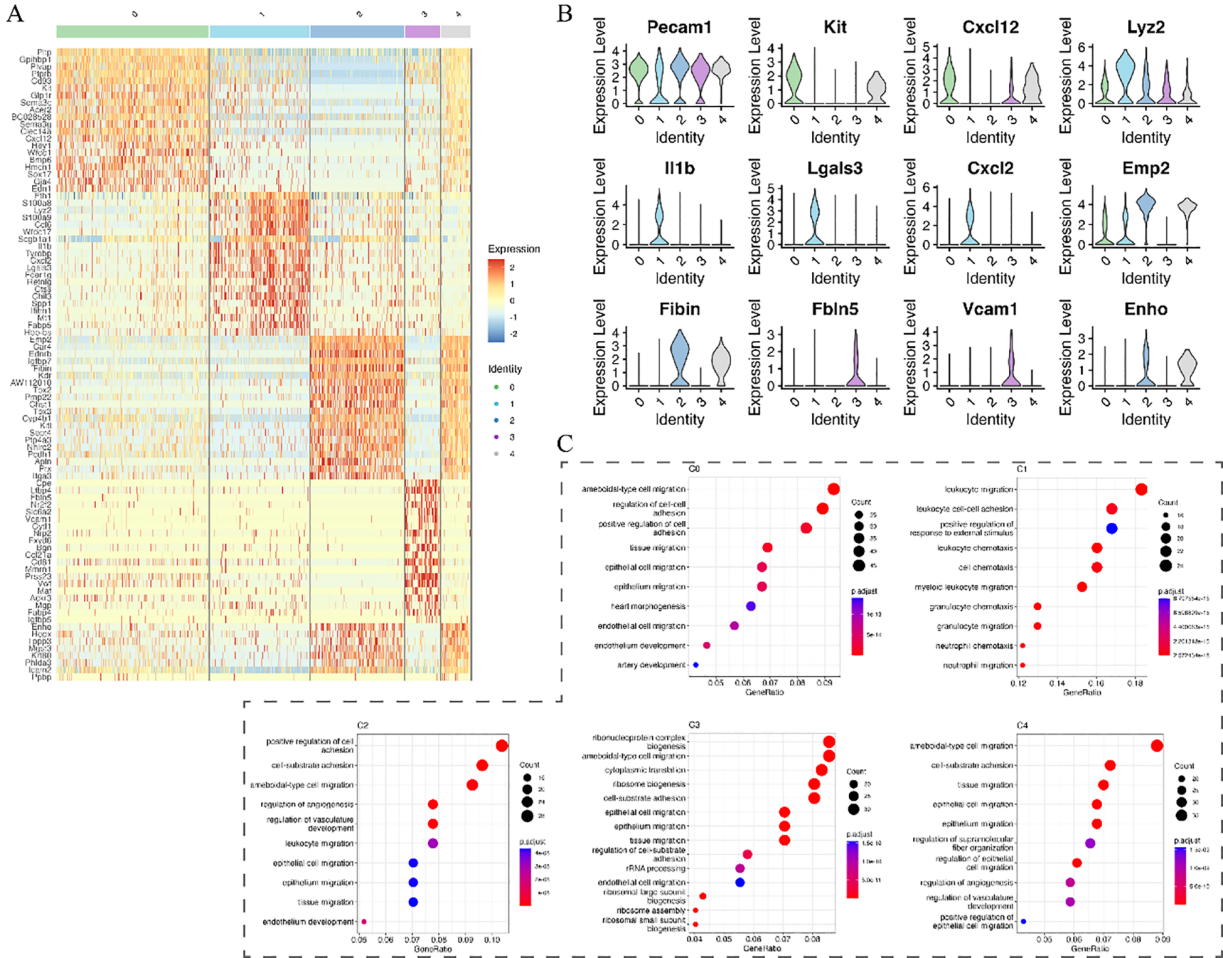
Marker gene expression and functional enrichment analysis of endothelial subpopulations. **(A)** Heatmap showing marker gene expression across endothelial subpopulations. **(B)** Violin plots of selected marker genes across different subpopulations. **(C)** KEGG pathway enrichment analysis highlighting biological processes associated with each subpopulation.

Marker gene analysis further clarified subpopulation characteristics. The progenitor-like C0 subpopulation, characterized by Kit expression, showed enrichment for endothelial development and migration pathways. Subpopulation C1 predominantly expressed immune-related genes involved in neutrophil chemotaxis (e.g., Il1b, Vcam1), whereas C2 exhibited gene signatures associated with angiogenesis and tissue repair (**Figures 5A–C**).

### 3.4 Endothelial cells contribute to the inflammatory response by promoting neutrophil migration

**Figure 6:**
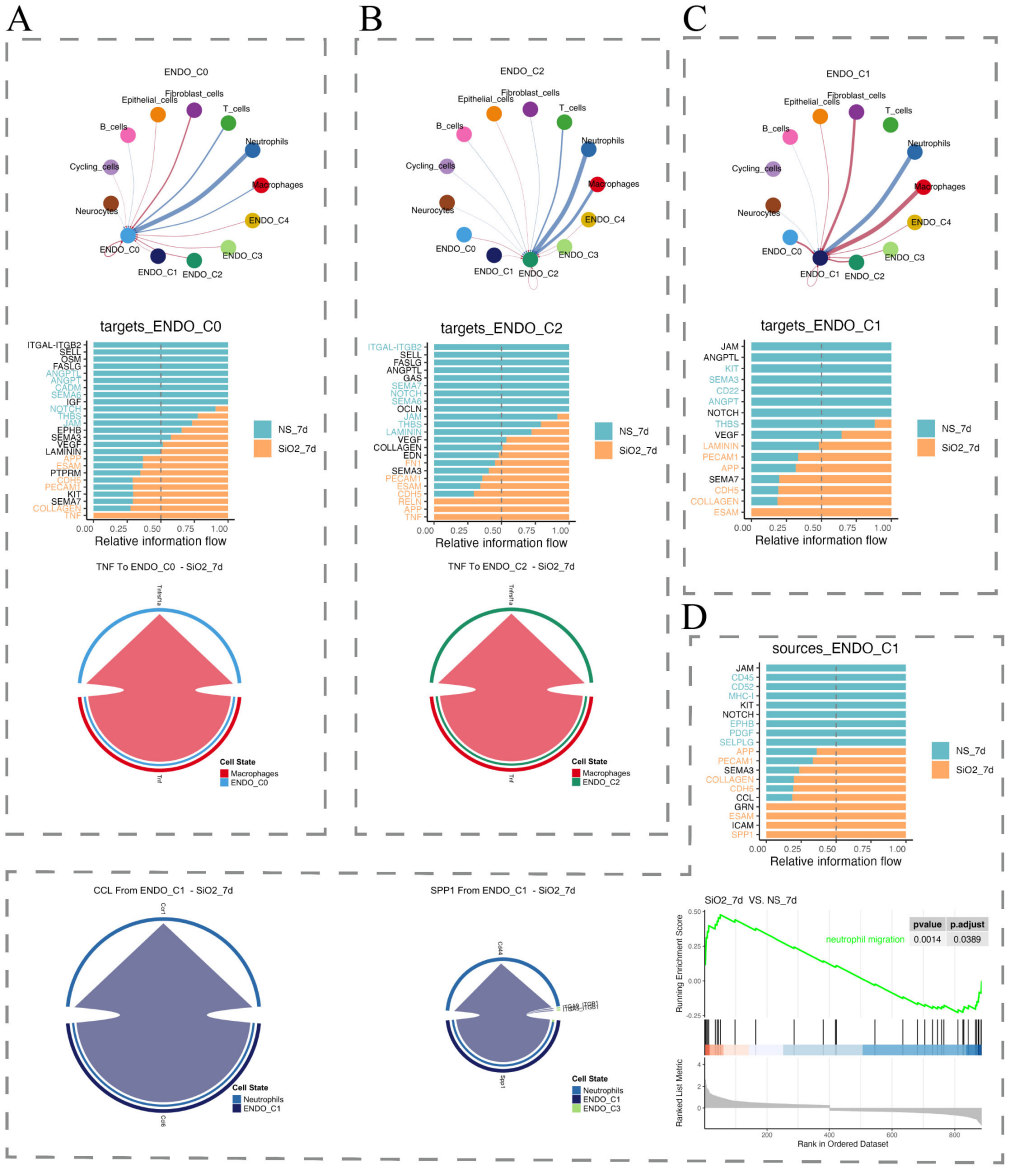
Cell-cell communication analysis of endothelial subpopulations. **(A–C)** Interaction networks of endothelial subpopulations (C0, C1, C2) with other cell types, along with relative information flow comparisons between control and disease groups. **(D)** Analyses of C1 subpopulation communication, including relative information flow (top) and GSEA of neutrophil migration-related processes (bottom).

To elucidate the interaction between endothelial cells and immune cells during silicosis, we utilized CellChat to analyze cell-cell communication. At 7 days post-exposure, C1 endothelial cells predominantly communicated through signaling interactions mediated by ESAM and CCL family proteins, and SPP1 pathways with neutrophils and macrophages, enhancing neutrophil recruitment (**Figures 6C, D**). Conversely, the C0 and C2 subpopulations were regulated primarily through TNF signaling from macrophages, indicating macrophage-mediated modulation of endothelial cell function (**Figures 6A, B**).

**Figure 7:**
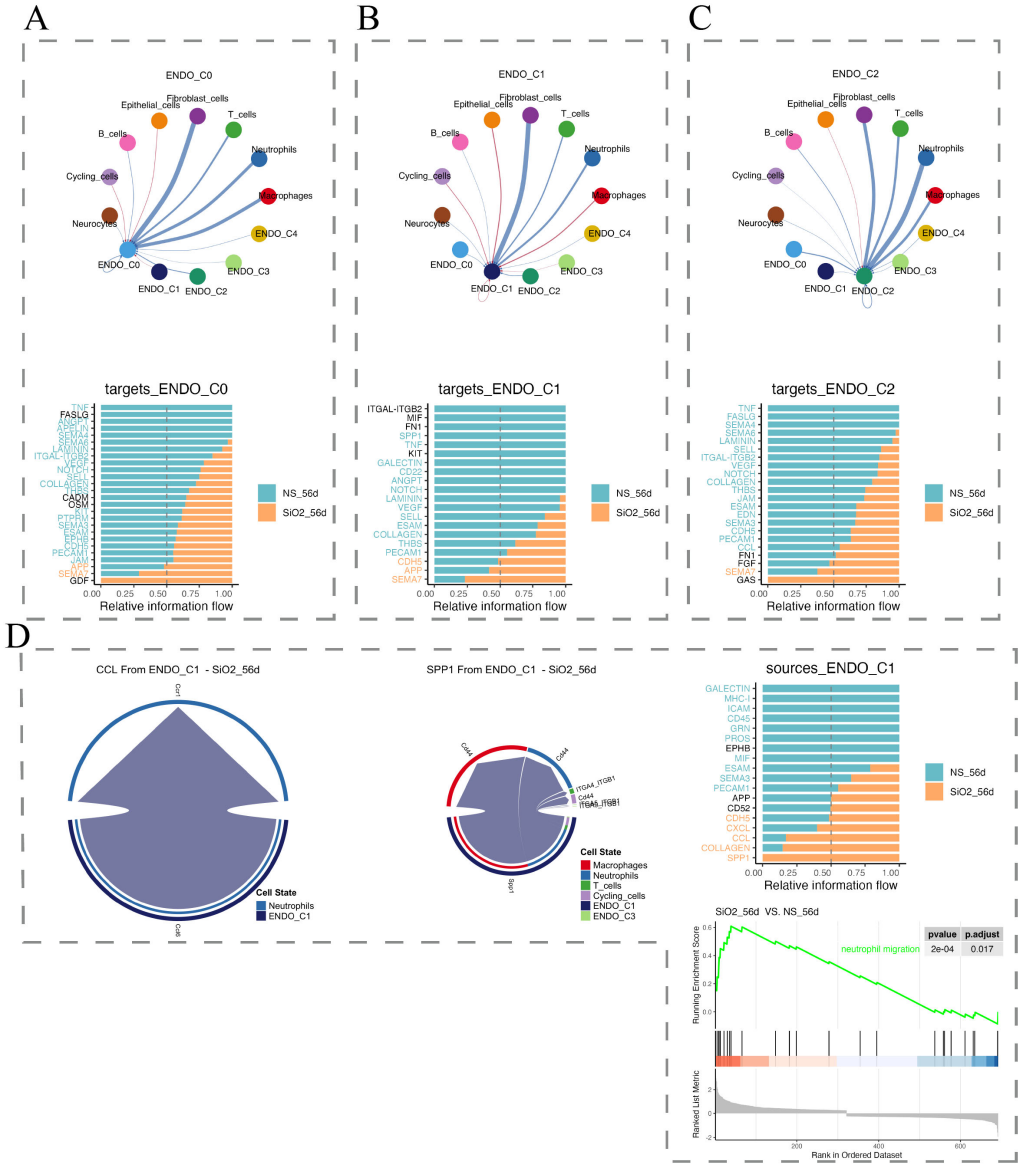
Cell-cell communication analysis in the 56d group. **(A–C)** Interaction networks of endothelial subpopulations (C0, C1, C2) with other cell types, along with relative information flow comparisons between control and disease groups. **(D)** Analyses of C1 subpopulation communication.

By day 56, C1 endothelial cells sustained pro-inflammatory signaling via SPP1 and CCL pathways, actively regulating macrophage and neutrophil activities, reinforcing persistent inflammation (**Figures 7B–D**). In contrast, C0 and C2 subpopulations showed unique activation of GDF (growth differentiation factor) and GAS (growth arrest-specific protein) pathways, indicating attempts at repair and regeneration (**Figures 7A, B**). Thus, endothelial subpopulations orchestrate complex responses during silicosis, simultaneously contributing to inflammation and attempting tissue repair.

Collectively, these findings indicate that endothelial cells significantly contribute to silicosis progression, predominantly through promoting sustained inflammation while concurrently attempting tissue repair. These insights have important implications for the development of targeted therapeutic strategies.

## 4 Discussion

Silicosis is one of the most severe occupational diseases worldwide, especially prevalent in developing countries. Its complex pathogenesis and lack of specific therapeutic strategies have resulted in persistently high morbidity and mortality rates, making it a significant public health issue (7). Fundamentally, silicosis is a pulmonary fibrotic disease characterized by dysregulated tissue repair responses, leading to excessive deposition of extracellular matrix (8). As a major component of the pulmonary vascular endothelium, endothelial cells have long been hypothesized to play a critical role in pulmonary fibrosis due to their spatial adjacency to epithelial and mesenchymal cells (9). This role is likely mediated through interactions involving multiple tissue cells, immune cells, and cytokines, forming a complex signaling network (10).

To further investigate the role of endothelial cells in silicosis, this study analyzed scRNA-Seq data from lung tissues of SiO2-induced mouse silicosis models. Previous studies have shown that inflammatory mediators released by macrophages in silicosis models can impair endothelial cell proliferation, migration, and repair functions (11). Our biological process analysis similarly identified a negative correlation between macrophages and endothelial cell proliferation, consistent with earlier findings. However, cell proportion analysis revealed that the proportion of endothelial cells was higher in the silicosis group than in the control group at both early and late stages, suggesting the existence of endothelial cell heterogeneity, with certain functional subpopulations potentially being activated under disease conditions.

Secondary dimensionality reduction of endothelial cells identified five subpopulations (C0 to C4). Subpopulations C0, C2, and C4 exhibited characteristic genes enriched in pathways associated with endothelial cell function, likely representing different stages of angiogenesis and repair. The C0 subpopulation, characterized by Kit+ progenitor cells, was enriched in pathways related to endothelial development and migration, potentially participating in early vascular regeneration and the initial stages of vascular repair in silicosis. C2 and C4 subpopulations were enriched in pathways related to cell motility and angiogenesis regulation, suggesting involvement in later stages of vascular regeneration, tissue repair, and remodeling of the inflammatory microenvironment. By contrast, the C1 subpopulation showed enrichment in immune cell migration pathways, likely representing inflammation-activated endothelial cells that play a central role in local inflammatory responses by promoting leukocyte transmigration and regulating the pulmonary inflammatory microenvironment. The C3 subpopulation, characterized by high metabolic activity, may represent a stress response to the inflammatory and repair microenvironments, participating in extracellular matrix remodeling or repair regulation.

Pseudotime trajectory analysis further revealed two major differentiation fates of endothelial cells. Fate 1, dominated by C1 and C3, exhibited high inflammatory and metabolic activity, corresponding to the persistent inflammation and tissue injury observed in silicosis progression. Fate 2, dominated by C2 and C4, showed enhanced angiogenic and repair capabilities, possibly reflecting the microenvironment’s attempt to reverse pathological states. These two fates form a dynamic competition between inflammation exacerbation and tissue repair.

Further cell-cell communication analysis revealed that in silicosis, immune cells persistently release cytokines such as TNF and IFN-γ, which act on the C0 and C2 subpopulations through related signaling pathways, causing sustained damage, inducing specific apoptosis, and inhibiting angiogenesis (12, 13). The inflammatory environment alters the number and molecular context of cell-cell interactions, disrupting spatial cell distribution, which partially explains the observed decline in cell communication in the disease group. Notably, during the fibrotic phase, the GDF pathway was activated in c-Kit+ endothelial progenitor cells (C0), potentially contributing to inflammation suppression and homeostasis maintenance (14, 15). Our previous study demonstrated that silica exposure induces a significant, time-dependent increase in pulmonary neutrophils, as confirmed by MPO immunohistochemical staining. The neutrophils were widely dispersed across the lung and accumulated particularly around the trachea and in the core areas of silicotic nodules. This indicates that neutrophil accumulation and NETs release play a crucial role in driving silica-triggered lung inflammation and fibrotic progression (16). Neutrophils actively participated in the inflammatory response by releasing inflammatory mediators. The immune-related C1 subpopulation persistently transmitted signaling molecules, such as CCL, SPP1, ICAM, and ESAM, during both the inflammatory and fibrotic phases. These pathways, some of which have been proven to be closely related to fibrosis progression (17), mediated neutrophil recruitment, adhesion, transmigration, and migration through endothelial cell-neutrophil interactions (18–23), thereby promoting the progression of silicosis.

In conclusion, this study demonstrates that endothelial cells exhibit two distinct differentiation fates during the progression of silicosis, forming a dynamic balance between inflammation exacerbation and tissue repair. The immune-related C1 subpopulation exacerbates the inflammatory environment by promoting neutrophil migration. Anti-inflammatory drugs, metabolic inhibitors, or pro-angiogenic therapies targeting these differentiation processes represent promising strategies for future silicosis treatment.

## Data Availability

Publicly available datasets were analyzed in this study. This data can be found here: GSE183682.

## Supplementary material

Supplementary Table 1 Proportions of major cell types across different groups. The table presents the relative proportions of macrophages, neutrophils, T cells, endothelial cells, fibroblasts, epithelial cells, B cells, cycling cells, and neurocytes in NS_7d, NS_56d, SiO_2__7d, and SiO_2__56d groups.

Supplementary Table 2 Proportions of endothelial cell subpopulations across different groups. The table shows the distribution of endothelial subpopulations (C0–C4) in NS_7d, NS_56d, SiO_2__7d, and SiO_2__56d groups.

Supplementary Figure 1 Pseudotime trajectory and marker gene expression analysis of endothelial cells. **(A)** Pseudotime analysis showing endothelial cell differentiation across subpopulations. **(B)** Expression trends of key marker genes along pseudotime. **(C)** Heatmap of dynamically expressed genes during endothelial differentiation.

Supplementary Figure 2 Cell-cell communication analysis. **(A, C)** Number and strength of interactions in 7d and 56d groups. **(B, D)** Network diagrams of cell-cell interactions in control and disease groups.
