# Supplementary material for: Single-cell transcriptomics reveals predominantly inflammatory endothelial cell responses and suppressed vascular repair in silicosis
Source: Front Immunol. 2025 Sep 3;16:1629226. doi: 10.3389/fimmu.2025.1629226 (PMC12440756; doi:10.3389/fimmu.2025.1629226)
Supplement: Supplementary Table 1 — Proportions of major cell types across different groups. The table presents the relative proportions of macrophages, neutrophils, T cells, endothelial cells, fibroblasts, epithelial cells, B cells, cycling cells, and neurocytes in NS_7d, NS_56d, SiO2_7d, and SiO2_56d groups. [file Table1.docx]

# Table

Table S1. Proportions of major cell types across different groups.

|  | NS_7d | NS_56d | SiO2_7d | SiO2_56d |
| --- | --- | --- | --- | --- |
| Macrophages | 0.324015 | 0.3535 | 0.497736 | 0.45285 |
| Neutrophils | 0.377607 | 0.223664 | 0.200362 | 0.17513 |
| T_cells | 0.192207 | 0.195929 | 0.162592 | 0.073575 |
| Endothelial_cells | 0.024334 | 0.058935 | 0.041262 | 0.063372 |
| Fibroblast_cells | 0.019699 | 0.049765 | 0.034407 | 0.064169 |
| Epithelial_cells | 0.009994 | 0.049877 | 0.012029 | 0.101873 |
| B_cells | 0.040846 | 0.05066 | 0.02975 | 0.031168 |
| Cycling_cells | 0.00956 | 0.013532 | 0.019273 | 0.033719 |
| Neurocytes | 0.001738 | 0.004138 | 0.002587 | 0.004145 |

Table S2. Proportions of endothelial cell subpopulations across different groups.

|  | NS_7d | NS_56d | SiO2_7d | SiO2_56d |
| --- | --- | --- | --- | --- |
| 0 | 0.440476 | 0.364326 | 0.404389 | 0.348428 |
| 1 | 0.107143 | 0.218216 | 0.141066 | 0.328302 |
| 2 | 0.309524 | 0.258065 | 0.250784 | 0.179874 |
| 3 | 0.089286 | 0.074004 | 0.147335 | 0.069182 |
| 4 | 0.053571 | 0.085389 | 0.056426 | 0.074214 |
