# Supplementary figures and images for: Single-cell transcriptomics reveals predominantly inflammatory endothelial cell responses and suppressed vascular repair in silicosis
Source: Front Immunol. 2025 Sep 3;16:1629226. doi: 10.3389/fimmu.2025.1629226 (PMC12440756; doi:10.3389/fimmu.2025.1629226)

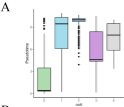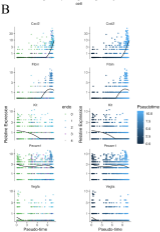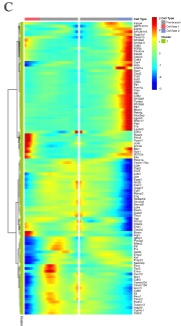

Supplement: Supplementary Figure 1 — Pseudotime trajectory and marker gene expression analysis of endothelial cells. (A) Pseudotime analysis showing endothelial cell differentiation across subpopulations. (B) Expression trends of key marker genes along pseudotime. (C) Heatmap of dynamically expressed genes during endothelial differentiation. [file DataSheet1.pdf]

A

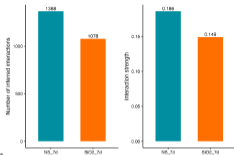

B

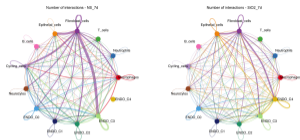

C

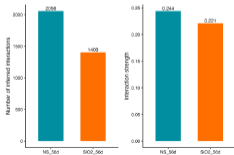

D

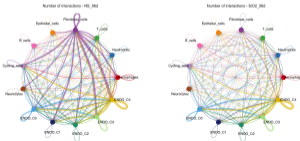

Supplement: Supplementary Figure 2 — Cell-cell communication analysis. (A, C) Number and strength of interactions in 7d and 56d groups. (B, D) Network diagrams of cell-cell interactions in control and disease groups. [file DataSheet2.pdf]
